# Modeling of Internal Geometric Variability and Statistical Property Prediction of Braided Composites

**DOI:** 10.3390/ma15155332

**Published:** 2022-08-03

**Authors:** Wenli Li, Donghui Zhu, Wenqi Shao, Dong Jiang

**Affiliations:** School of Mechanical and Electronic Engineering, Nanjing Forestry University, Nanjing 210037, China; liwenli@njfu.edu.cn (W.L.); zhudonghuinjfu@outlook.com (D.Z.); vickie0626@outlook.com (W.S.)

**Keywords:** braided composite, equivalent elastic parameter, thermal expansion coefficient, uncertainty modeling, Monte-Carlo simulation

## Abstract

Due to the advantages of high specific strength, specific stiffness, and excellent fatigue resistance, carbon fiber reinforced braided composites have been widely applied in engineering. Since the molding process of braided composites is complex and immature, substantial variability of the internal geometry exists in composites, in which the yarn path with uncertainty is a main factor, so it is necessary to establish an uncertainty model to study the influence of randomness of the yarn path on mechanical properties, which is significantly related to the fatigue resistance properties of composite. An uncertain mesoscopic model with uniform distribution of yarn paths is proposed. Assuming the yarn path is spatially varying in interval range, the variability of yarn path is represented geometrically in the unit cell of composite. The three-dimensional coordinates of the yarn trajectory are calculated, the meso-uncertainty models of 2-D and 2.5-D braided composites are established. The equivalent elastic parameters and the thermal expansion coefficients are obtained by applying homogenization method and temperature field boundary conditions to the mesoscopic model. The effect of yarn path uncertainty on the statistical characteristics of elastic and thermal parameters of braided composites was studied by using Monte-Carlo simulation. A simulation method for modeling yarn path uncertainty of braided composites is provided in this paper for predicting the statistical characteristics of the equivalent elastic and thermal parameters.

## 1. Introduction

Braided composites have been widely used in aerospace due to their excellent properties, such as high stiffness, high strength to weight ratio, and good fatigue strength [1,2,3]. They can reduce the use of high alloy on the aircraft, save on structural costs, and have a great advantage in weight reduction and efficiency. The research on composite materials is usually based on the elastic stage, and then further study on the mechanical properties of materials in the plastic stage, impact, strength, fatigue, and other load conditions. To make more efficient use of braided composites, we need to further study their mechanical properties.

Obtaining the elastic constants of braided composites is one of the important topics in research. Based on the homogenization theory, 3D composite materials are equivalent to orthotropic materials with 9 independent parameters. Accurate parameters are the basis for studying the mechanical properties of composites [4,5]. However, the yarn path is uncertain due to the complexity of the braided composite forming process. To improve the reliability of numerical analysis of braided composite structures, it is necessary to study the effect of randomness on mechanical properties of composites [6,7,8,9].

In geometric modeling of braided composites, yarn paths are usually represented by specific function curves, which makes modeling difficult [10,11,12]. Tsai K H [13] used one-dimensional spring units to simulate yarn predicts elastic properties. However, the actual yarn material parameters are three-dimensional, so the simulation is not accurate enough. In whole mesoscopic modeling, the cubic B-spline curve fits the position points of the sand carrier to obtain a more real yarn path. Kai [14] uses cubic B-spline curves to fit the relatively real position of the yarn path. Kai’s conclusion is that the full-size model can produce more accurate equivalent results than the mesoscopic model, but the mesoscopic model has faster calculation time and higher efficiency.

In addition, obtain the equivalent elastic parameters is an essential step to establish the model. The prediction of equivalent elastic modulus and thermal expansion of braided composites has been widely studied [15,16]. Although some uncertain parameters of composite materials can be measured by experimental tests [17,18,19], these methods are often expensive, time-consuming and inefficient. Hill [20] first proved that the strain energy of homogenized macro continuum is equal to that of microscale unit cell from the strain energy perspective. The regular and representative volume elements of braided composites are analyzed, and the unit cell model reflecting the parameters of composites such as component information and braided form is extracted. The elastic parameters can also be obtained using the homogenization method. Therefore, specific periodic displacement boundary conditions or traction conditions can be applied to obtain the stress distribution [21], and then the equivalent stiffness can be obtained through the average stress and strain in the constitutive equation. Xia [22] applied displacement boundary conditions to the unit cells with parallel opposite edges, which could satisfy the continuous displacement and stress of the periodic structure. Hiroshi [23] derived two equations based on homogeneous method of boundary conditions and weighted residual theory, and obtained equivalent parameters. After predicting stiffness with periodic boundary conditions, Chen [24] uses the dynamic characteristic error between the refined model and the homogeneous model to illustrate the accuracy of prediction. The stiffness obtained from the multi-scale model above is based on statics, so the model can be further used as the basis for strength analysis in terms of stress analysis [25]. Most studies verify the accuracy of strength prediction of the multi-scale model combined with tests [26,27].

Common multi-scale modeling software includes Digimat [28], WiseTex [29], and TexGen [30]. Among them, TexGen is an open source software developed by the composite Materials Research Group of the University of Nottingham for geometric modeling of textile structures [31], connected with ABAQUS finite element analysis software. This software is the basis for studying the mechanical properties of braided composites [32].

In this study, an uncertain mesoscopic model of braided composite yarn paths is proposed, which follows an interval uniform distribution. Using the combination of simulation software and finite element analysis software, the meso-uncertainty model can be established quickly, and the mechanical properties of composites can be studied more efficiently. The outline of the work is as follows: Section 2 describes the main research methods and processes of this paper. In Section 3.1, modeling of 2d braided composites and the effect of yarn path uncertainty on thermoelastic parameters were studied by equivalent method. In Section 3.2, a mesoscopic model of 2.5D braided composites was established, and the effect of yarn path uncertainty on equivalent thermoelastic parameters of braided composites was studied by means of 1000 equivalence.

## 2. Methodology

Based on the research literature, a mesoscopic model with uncertain yarn path is proposed. Si/C matrix and Toray T300-1K fiber bundle are used in this model. It is an orthotropic material with nine independent parameters. Assuming the yarn path is spatially varying in an interval range, the variability of yarn path is represented geometrically in the unit cell of composite. First, load boundary conditions are applied to the model to obtain equivalent parameters. Then, the yarn path uncertainty is equivalent and the interval uniform distribution is assumed. Finally, the influence of yarn path change on elastic parameters and thermal expansion coefficient was obtained using finite element analysis software.

Conventional finite element modeling software generally has tetrahedron and hexahedron meshes. In addition, Voxel, DST, MFEM, and XFEM meshes can be used to quickly establish finite element models. The Voxel method is used in Texgen to discretely establish the meso model of braided composites. It has two main algorithms to avoid intrusions between yarns. The first method is to determine whether the points on the yarn’s surface are contained in any other yarn, but this method does not work if there are not enough points. The second is to judge whether the envelope of each yarn surface intersects.

**Figure 1:**
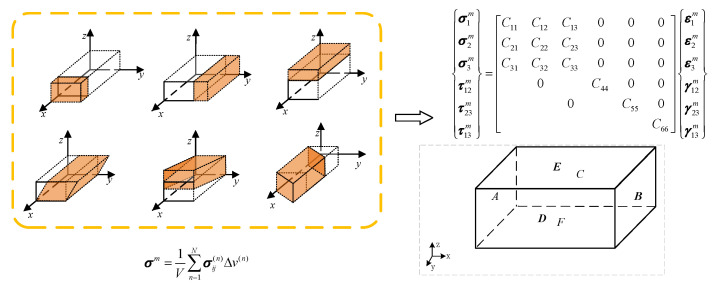
Schematic diagram of boundary conditions of the unit cell model.

It is assumed that the macroscopic mechanical properties of braided composites are orthogonal anisotropy and have three orthogonal symmetry planes. The left and right, back and front, and upper and lower planes are labeled as planes A, B, C, D, E and F. The constitutive relation of orthotropic composites can be expressed in Figure 1.

Taking the displacement boundary conditions in the *x* direction as an example, plane A is subjected to A fixed constraint (0, 0, 0), and plane B is subjected to A displacement in the *x* direction (*x*_1_, 0, 0). The freedom of other planes in the *x* direction is released, and the constraints in the *y* and *z* directions are (_, 0, 0). The strain component in the constitutive equation is [*ε*_1_*^m^* = 1, 0, 0, 0, 0]. Then the first column elements of the stiffness matrix are obtained by means of the stress average method. The other aspects are shown in Table 1.

The main implementation steps of the method are as follows:

TexGen textile material simulation software was used to efficiently study the effect of yarn path uncertainty on the mechanical properties of mesoscopic structures. Due to the need to establish a mesoscopic model of uncertainty [33], according to the help document, Python script language was used to establish 2D and 2.5D mesoscopic uncertainty models of braided composite materials, and equivalent elastic parameters could be obtained based on the homogenization theory.

(1) The three-dimensional coordinates of the yarn paths are numerically calculated, and the coordinate values are obtained by cubic B-spline curve interpolation to obtain smooth curve coordinates.

(2) The equivalent stiffness is obtained according to the homogenization theory, and the uncertainty model is established without considering the special probability distribution of the characteristic quantity.

(3) The equivalent elastic parameters and thermal expansion coefficient are obtained by applying the boundary conditions of concentrated force and temperature field load to the model. Monte Carlo equivalent method was used to obtain the effect of uncertainty on the thermoelastic parameters of braided composites.

### Obtaining Equivalent Parameters by Loading Boundary Conditions

**Figure 2:**
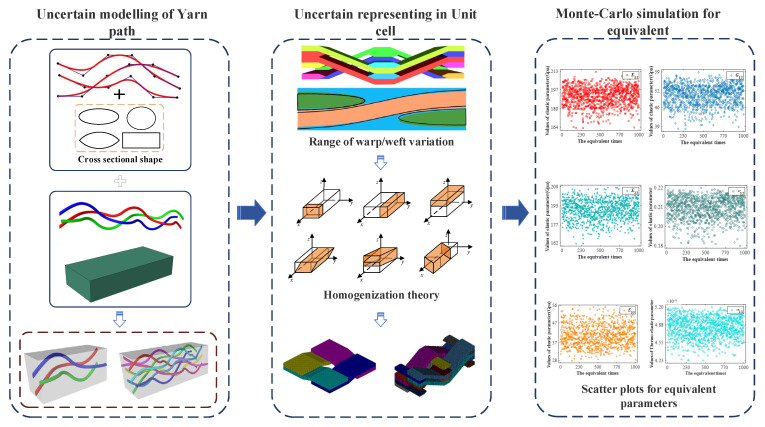
The process of equivalent modeling analysis.

Composite material is composed of multiple components, and the non-uniform continuum is represented by *M*, as shown in Figure 2. According to the knowledge of solid mechanics, this heterogeneous continuum satisfies the equilibrium equation and the motion equation in the *R* domain:

(1) $$\begin{array}{l} \sigma_{ij,j}+{\bar{f}}_{i}=\rho \cdot {\ddot{u}}_{i}\;\;\;\;in\;R\\ \sigma_{ij}=C_{ijkl}\cdot \varepsilon_{kl} \end{array}$$

*σ_ij_* and *ε_kl_* are stress and strain tensors, $\bar{f}$ is the strength received in the *R* domain, *ρ* is the volume density, and *C_ijkl_* is the elastic tensor of the constitutive equation. On the boundary *S_σ_* and *S_u_*, the continuum satisfies the following equation under the basic and natural boundary conditions:

(2) $$\begin{array}{ll} t_{i}=\sigma_{ij}n_{j}={\bar{t}}_{i} & \;\;on\;S_{\sigma }\\ u_{i}={\bar{u}}_{i} & \;\;on\;S_{u} \end{array}$$

**Figure 3:**
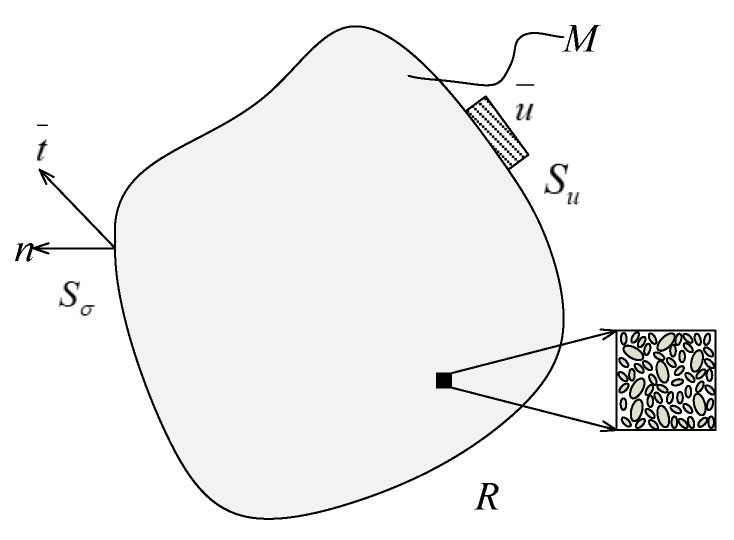
Boundary conditions for heterogeneous material M in R domain.

In Figure 3, *n_j_* is the unit vector perpendicular to the *S_σ_* boundary, ${\bar{t}}_{i}$ is the traction. The key to realize the homogenization is to obtain the equivalent stiffness of the composite. For the periodic continuum structure, the uniform displacement boundary conditions can ensure that the displacement of the whole structure is continuous, and the distribution of traction force at the relative parallel boundary is the same. Therefore, combined with the above formula, the problem of obtaining macroscopic global elastic parameters of reactive periodic structures from the unit cell model can be solved by applying specific displacement boundary conditions and concentrating forces.

Hill [19] pointed out from the strain energy perspective that when given the boundary condition *u*, *σ_ij,j_* = 0 in the volume of a unit cell. The average strain energy in the region can be obtained from the average stress and strain. Similarly, it can be proved that the average strain energy can be obtained when the boundary is subjected to a traction which produces non-uniform internal stress in the material.

(3) $$\sigma^{m}\cdot \varepsilon^{m}=\frac{1}{\left|V\right|}\int_{V}\sigma \cdot \varepsilon dV$$

Formula (3) is Hill energy averaging theorem, which proves that the strain energy of homogenized macro continuum is equal to that of microscale unit cell. *σ^m^* and *ε^m^* are macroscopic stress and strain tensors. *σ* and *ε* are the stress and strain tensors of unit cell, respectively.

When a force is applied in each direction of a unit cell, the following relation can be obtained:

(4) $$\begin{array}{l} \sigma_{1}^{m}=F_{1}/V,\;\sigma_{2}^{m}=F_{2}/V,\;\sigma_{3}^{m}=F_{3}/V,\\ \tau_{23}^{m}=F_{23}/V,\;\tau_{31}^{m}=F_{31}/V,\;\tau_{12}^{m}=F_{12}/V. \end{array}$$

*V* is the volume of a unit cell. The macroscopic stress can be expressed by the force applied to the unit cell model and the equivalent material parameters can easily be expressed by the unit cell strain degree of freedom *ε*_1_*^m^*, *ε*_2_*^m^*, *ε*_3_*^m^*, *γ^m^*_23_, *γ^m^*_31_, *γ^m^*_12_, and the loads are represented by *F*_1_, *F*_2_, *F*_3_, *F*_23_, *F*_31_, *F*_12_, Δ*T*. The coefficient of thermal expansion can be obtained by applying a specific temperature load to the mesoscopic model. The equation for the relationship between orthogonal anisotropy parameters and loads is listed below.

*E*_1_, *E*_2_, *E*_3_ meets the following conditions:

(5) $$\begin{array}{l} E_{1}^{m}=\sigma_{1}^{m}/\varepsilon_{1}^{m}=F_{1}/V\varepsilon_{1}^{m}\\ \;When\;F_{1}\neq 0,\;F_{2}=F_{3}=F_{23}=F_{31}=F_{12}=0;\\ E_{2}^{m}=\sigma_{2}^{m}/\varepsilon_{2}^{m}=F_{2}/V\varepsilon_{2}^{m}\\ \;When\;F_{2}\neq 0,\;F_{1}=F_{3}=F_{23}=F_{31}=F_{12}=0;\\ E_{3}^{m}=\sigma_{3}^{m}/\varepsilon_{3}^{m}=F_{3}/V\varepsilon_{3}^{m}\\ \;When\;F_{3}\neq 0,\;F_{1}=F_{2}=F_{23}=F_{31}=F_{12}=0; \end{array}$$

*G*_23_, *G*_31_, *G*_12_ meets the following conditions:

(6) $$\begin{array}{l} G_{23}^{m}=\tau_{23}^{m}/\gamma_{23}^{m}=F_{23}/V\gamma_{23}^{m}\\ \;When\;F_{23}\neq 0,\;F_{1}=F_{2}=F_{3}=F_{31}=F_{12}=0;\\ G_{31}^{m}=\tau_{31}^{m}/\gamma_{31}^{m}=F_{31}/V\gamma_{31}^{m}\\ \;When\;F_{31}\neq 0,\;F_{1}=F_{2}=F_{3}=F_{23}=F_{12}=0;\\ G_{12}^{m}=\tau_{12}^{m}/\gamma_{12}^{m}=F_{12}/V\gamma_{12}^{m}\\ \;When\;F_{12}\neq 0,\;F_{1}=F_{2}=F_{3}=F_{23}=F_{31}=0; \end{array}$$

*v*_12_, *v*_23_, *v*_31_ meets the following conditions:

(7) $$\begin{array}{l} \nu_{12}^{m}=-\varepsilon_{2}^{m}/\varepsilon_{1}^{m}\\ \;When\;F_{1}\neq 0,\;F_{2}=F_{3}=F_{23}=F_{31}=F_{12}=0;\\ \nu_{23}^{m}=-\varepsilon_{3}^{m}/\varepsilon_{2}^{m}\\ \;When\;F_{2}\neq 0,\;F_{1}=F_{3}=F_{23}=F_{31}=F_{12}=0;\\ \nu_{31}^{m}=-\varepsilon_{1}^{m}/\varepsilon_{3}^{m}\\ \;When\;F_{3}\neq 0,\;F_{1}=F_{2}=F_{23}=F_{31}=F_{12}=0; \end{array}$$

*𝛼*_1_, *𝛼*_2_, *𝛼*_3_ meets the following conditions:

(8) $$\begin{array}{l} \alpha_{1}^{m}=\varepsilon_{1}^{m}/\Delta T\\ \;When\;F_{1}=F_{2}=F_{3}=F_{31}=F_{23}=F_{12}=0;\\ \alpha_{2}^{m}=\varepsilon_{2}^{m}/\Delta T\\ \;When\;F_{1}=F_{2}=F_{3}=F_{31}=F_{23}=F_{12}=0;\\ \alpha_{3}^{m}=\varepsilon_{3}^{m}/\Delta T\\ \;When\;F_{1}=F_{2}=F_{3}=F_{31}=F_{23}=F_{12}=0; \end{array}$$

When obtaining each of these attributes, the corresponding load conditions must be satisfied. It can be observed that if the composites are orthotropic, the above properties are sufficient to describe them in most cases. However, the orthogonal anisotropy equivalent parameters mentioned above will not be enough if irregular geometry or local microcracks are involved in the composite cells, i.e., the uncoupled parts of the cell produce shear stress and strain components, resulting in the general anisotropy of the material. For such materials, more material properties are needed to describe their mechanical properties.

## 3. Case Study

### 3.1. Mesoscopic Uncertainty Modeling of 2D Braided Composites

#### 3.1.1. Meso-Geometric Model of Plain Weave Composites

**Figure 4:**
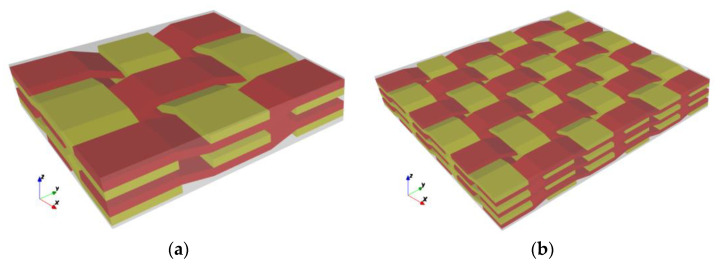
The parametric model of weave composite. (**a**) 3 × 3 × 2 geometric model, (**b**) 6 × 6 × 3 geometric model.

To further characterize the uncertainty of plain weave composite yarn amplitude, the number of warp and weft yarns is also taken as parameterized input, and the modeling idea is as follows: First, several weft yarns, which are uncertain in the *z* direction, are generated according to the number of warp yarns and weft yarns. Since warp and weft yarns fit each other, it is easier to obtain the corresponding coordinates of warp yarns and generate uncertainty modeling of plain weave composite materials. Figure 4 shows the established parameterized mesoscopic uncertainty parameterized model of plain weave composite material. Yellow is weft yarn, red is warp yarn, and each yarn fluctuates within a certain range.

**Figure 5:**
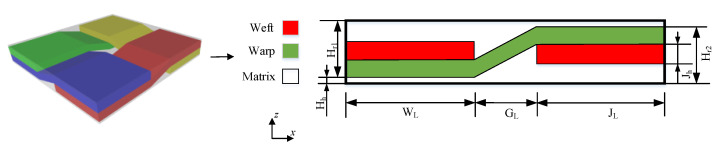
The uncertain geometry of weave composite.

There was serious matrix intrusion between yarns during modeling. Therefore, in this example, the yarn path follows uniform distribution changes in a certain range without considering the special probability distribution in the geometric model. In fact, there is a very small amount of matrix filling between weft and warp. Ideally, the unit cell model of plain weave composite is assumed to be the trig function of the weft path, and the warp cross section fits the contour of the trig function, but this problem is relatively complicated. In this section, the analysis model is simplified as shown in Figure 5, and the meso-uncertain geometric model of plain braided composite material is further established. To simplify the problem, the established model makes the following assumptions: (i) The yarn section is simplified to a rectangle; (ii) The yarn path is assumed to be a broken line; (iii) There is no matrix filling between yarns; (iv) It is only considered that the yarn amplitude in the plane follows uniform distribution, yarn torsion, deformation in other directions and defects are not considered.

According to Ref. [34], the geometric parameter design description of the uncertainty model of plain braided composite material and the values of the model in this paper are shown in Table 2. The geometric characteristics of the yarn are parameterized as input. Where *J_L_*, *J_h_*, *W_L_* and *W_h_* are the length and width of the rectangular warp and weft sections respectively. *G_L_* is the distance between two adjacent yarns. The height of yarn is uniformly distributed within the interval [0,0.028]. The distance between yarn and outer matrix is constant value 0.04.

#### 3.1.2. Equivalent Results

**Figure 6:**
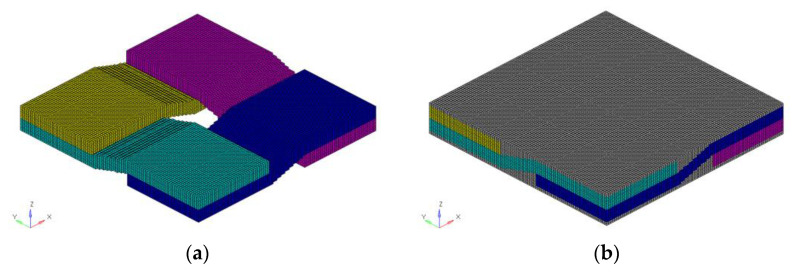
The finite element model for weave composite. (**a**) Mesoscopic model without considering the matrix. (**b**) Mesoscopic model considering the matrix.

The fiber bundle used in the calculation example is Toray T300-1K with a density of 1.76 g/cm^3^. The matrix of composite material is Si/C material, and the elastic parameters of each component of braided composite material are shown in Table 3 and Table 4:

The finite element model of braided composite material established by *Voxel* technology is shown in Figure 6. The element divided by this method is C3R8, which is a regular grid of regular hexahedron element, and appropriate local mechanical properties and material direction are defined at the center of the element. The yarn simulated by this element is serrated, so the mesh density needs to be increased to describe the yarn path more accurately and reduce the influence of mesh shape on the equivalent result. The number of yarns was reduced to reduce the influence of independent variables on elastic parameter response, the numerical was 2 × 2. Although the parametric geometric model is established, the influence of the number of derived meshes on the computational efficiency still needs to be considered. The number of grids established after the above consideration is 104,400, and the number of nodes is 117,660. The volume fraction of carbon fiber in warp and weft yarn was 0.82%, the yarn accounted for 64.46% of the total volume, and the carbon fiber content in the model was about 50%.

**Figure 7:**
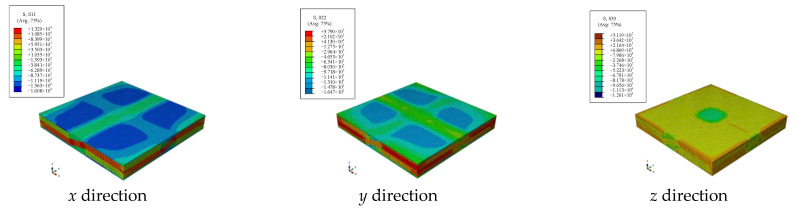
The stress distribution under load boundary conditions.

By applying load boundary conditions according to Equation (4), strain distributions of elements under six working conditions can be obtained as shown in Figure 7. According to the relationship between load and elastic parameters between degrees of freedom in Equations (5)–(8), equivalent parameters can be obtained.

According to the constitutive equation of stress-strain relationship, the equivalent stiffness matrix *C* can be obtained as follows:

(9) $$C=\left[\begin{array}{cccccc} 5.422\times 10^{-12} & -1.094\times 10^{-13} & -1.341\times 10^{-12} & 0 & 0 & 0\\ -1.094\times 10^{-13} & 5.388\times 10^{-12} & -1.365\times 10^{-12} & 0 & 0 & 0\\ -1.341\times 10^{-12} & -1.365\times 10^{-12} & 2.504\times 10^{-11} & 0 & 0 & 0\\ 0 & 0 & 0 & 7.696\times 10^{-11} & 0 & 0\\ 0 & 0 & 0 & 0 & 8.276\times 10^{-11} & 0\\ 0 & 0 & 0 & 0 & 0 & 2.091\times 10^{-11} \end{array}\right]$$

The flexibility matrix can be obtained by inverting the equivalent elastic matrix. According to the relationship between flexibility matrix and elastic parameters, the equivalent elastic parameters can be obtained. It can be seen from Table 5 that the arrangement of in-plane yarns in two directions is similar due to the structure of orthogonal plain weave composite material. Therefore, the equivalent elastic parameter *E*_11_ is close to *E*_22_.

#### 3.1.3. Equivalent Results of Yarn Path Uncertainty

**Figure 8:**
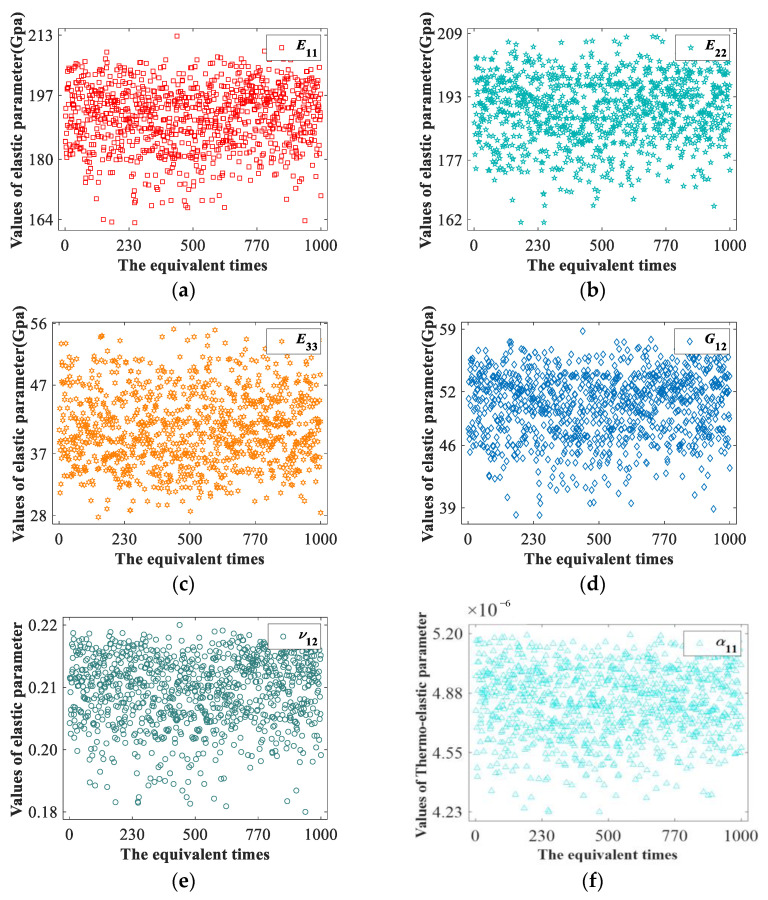
The scatter plots of equivalent results. (**a**) *E*_11_ Equivalent result scatter diagram. (**b**) *E*_22_ Equivalent result scatter diagram. (**c**) *E*_33_ Equivalent result scatter diagram. (**d**) *G*_12_ Equivalent result scatter diagram. (**e**) *ν*_12_ Equivalent result scatter diagram. (**f**) *α*_11_ Equivalent result scatter diagram.

It can be seen from Table 2 that the height difference between warp and weft yarns is between [0,0.028], and the distance between adjacent yarns is between [0,0.02]. The process of obtaining equivalent elastic parameters based on TexGen software and the equivalent results of elastic parameters *E*_11_, *E*_22_, *E*_33_, *G*_12_, *ν*_12_ and thermal expansion coefficient *α*_11_ were calculated by Abaqus software for 1000 s, as shown in Figure 8. As can be seen from the distribution of scattered points, the equivalent parameters will be affected to varying degrees as the yarn path changes within the interval. The total distance *R* of the elastic moduli of *E*_11_, *E*_22_ and *E*_33_ were 49.65 Gpa, 46.95 Gpa and 27.42 Gpa, respectively. The polar distance of Poisson’s ratio *ν*_12_ is 0.0368. The shear modulus *G*_12_ mainly varies in the range of 20.597 Gpa. R is the difference value between the maximum and minimum of the equivalent parameter.

The mean value only reflects the midpoint value of a set of data, while the mean square error can reflect the dispersion degree of a set of data. In this paper, the influence degree of yarn path on elastic parameters can be reflected side by side. According to the data in Table 6 and Table 7, it can be concluded that the influences on the elastic moduli *E*_11_ and *E*_22_ are relatively large. The standard deviation of *E*_33_ and *G*_12_ was 5.440 and 3.704, respectively. For the elastic parameters *ν*_12_, *ν*_23_, *ν*_31_, *G*_23_, *G*_31_ have relatively little effect. The pole distances of shear moduli *G*_23_ and *G*_31_ are relatively small around 10^3^ Mpa. In addition, the expansion coefficient of carbon fiber itself is small, so it has little influence on the thermal expansion coefficient. It can be concluded that the elastic parameters *E*_11_, *E*_22_, *E*_33_, and *G*_12_ are greatly affected when the yarn path of plain weave composite is changed.

For further analysis, the influence of yarn trend amplitude on elastic parameters. In this paper, the correlation coefficient between elastic parameters and fiber amplitude is solved, and the correlation coefficient between elastic parameters is given to study the quantitative variation relationship between elastic parameters. According Table 8, the correlation coefficients of elastic modulus, shear modulus and Poisson’s ratio of *ν*_12_ are all positive. The correlation coefficients of *E*_33_, *ν*_12_ and *ν*_23_ are all greater than 0.652, which indicates that these parameters are highly correlated. It shows that the above equivalent parameters increase or decrease together with the change in yarn path. Only Poisson’s ratio *ν*_23_, *ν*_31_ and other parameters appear negative correlation, and the correlation appears below 0.5.

### 3.2. Meso-Uncertainty Modeling of 2.5D Braided Composites

#### 3.2.1. 2.5D Meso-Geometric Model of Braided Composite Materials

Compared with plain weave, 2.5D braided composite has a more complex structure and mechanical mechanism, so further research is needed. To facilitate calculation, the uniform distribution of the weft in the *z* direction. When the warp yarn changes in the direction of height, the cross section also changes constantly. To ensure the fit of warp yarn, we determine the contour of the weft yarn according to the changing height of warp yarn.

**Figure 9:**
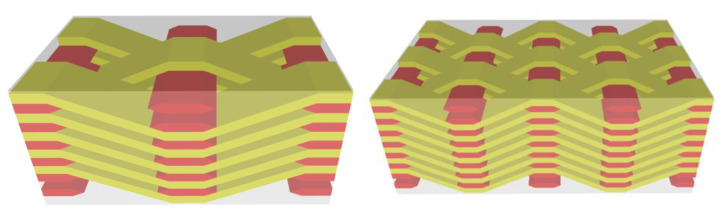
The micromodel of 2.5D braided composites.

In this paper, according to the number of weft yarns in *x* and *z* directions and warp yarns in *y* direction, a relatively simple mesoscopic parametric model of 2.5D braided composites was established to study the effects of yarn height fluctuation in *z* direction and transverse yarn distance on elastic parameters of 2.5D braided composites. Figure 9 shows the 2.5D weaving meso model, in which yellow is warp yarn and red is weft yarn.

This part is more difficult to achieve, is the use of MATLAB numerical calculation to ensure. In addition, the height of two adjacent warp yarns in the *y* direction is different, so the cross section of the weft yarns is certain. Parametric modeling is carried out in the *y* direction according to the number of warp yarns. After the above two steps of modeling, according to the number of *z* direction weft yarn, the lateral fluctuation of weft yarn is not considered. The warp yarn and weft yarn contact each other, layer upon layer of each other. To sum up, when the yarn position in *x* direction is determined, parametric modeling in the other two directions is equivalent to translating the yarn in each direction by a certain distance.

**Figure 10:**
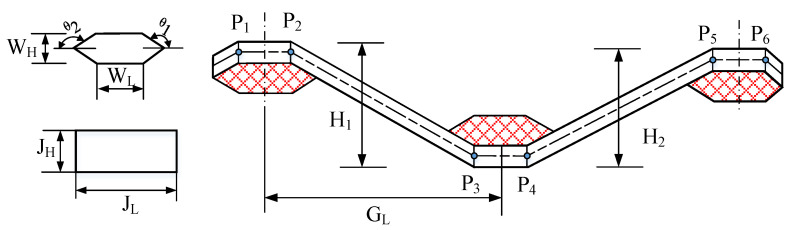
The uncertain geometry of 2.5D braided composite.

For the 2.5D braided composite with layer and shallow layer crossbending connection, the model is established as shown in Figure 10. The path is assumed to be a broken line to avoid the difficulty of modeling yarn as a curve. To simplify the modeling problem, the following assumptions are made: (i) the warp section is simplified as rectangle and the weft section as flat hexagon. (ii) the warp path is a broken line, the weft path is a straight line. (iii) Fit between yarns without matrix filling. (iv) Consider only the uniform distribution of the warps in the topper vertical direction, excluding the transversal fluctuations of the weft, deformations in other directions, and substrate defects. The yarns fit together without matrix filling which means that when the warp changes in height, the height of the filling changes with it. In addition, when the warp height of rectangular section changes, the inclination angle of the four edges of the filling hexagon should also change. Since the warp is a broken line, the slope can be found from the distance between its height and the warp. In addition, a weft is attached to two spatially intersecting warp yarns. P_1_–P_6_ are the intersections of the warps. Therefore, the dip Angle of the side edge of the weft section needs to consider the effect of the uncertainty of the two weft paths. When the transverse distance of the weft is constant, the slope of the four edges can be obtained according to the height of two warp yarns crossed in space. After a series of numerical operations to obtain the weft section of the profile and assigned to the yarn, this model can avoid overlapping area between warp and weft yarns and the uncontrollable shape of weft section. Finally, the uncertainty modeling of path height and spacing of 2.5D braided composite yarn was realized.

Description of geometric parameter design of 2.5D braided uncertainty model. Parameter values are shown in Table 9. The geometric features in the model are also taken as parameterized inputs to achieve uncertainty modeling. Where *J_L_* and *J_h_* are the length and width of the rectangular warp and the filling section, *W_L_* and *W_h_* are the length and height of the short side of the flat hexagon, respectively, and *G_L_* is the distance between the filling axes. The same height changes in uniform distribution between the interval [0,0.028]. The distance between the highest and lowest position of yarn and the outer matrix is constant value 0.04.

#### 3.2.2. Equivalent Results

**Figure 11:**
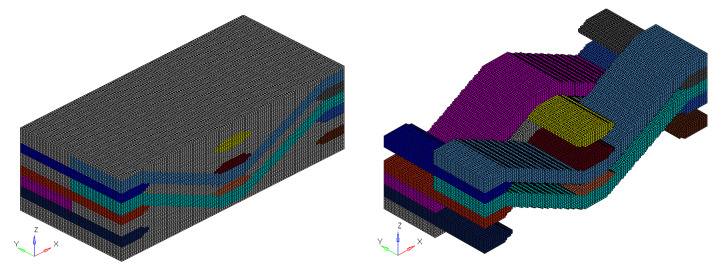
The finite element mesh for microstructure of 2.5D.

The time factor and the calculation efficiency of the finite element model still need to be considered. Figure 11 shows the finite element model of 2.5D braided composite. The composite material is the toray T300-3K fiber bundle. Its linear density is 198 g/km, and its density is 1.76 g/cm^3^. The material parameters of each component are the same as those in Table 3. The number of voxel grids in *x*, *y* and z directions is [150, 75, 75], the number of grids is 843,750, and the number of nodes is 872,176. The volume fraction of warp fiber is 69%, weft fiber is 82%, and the volume fraction of yarn of the whole model is 43.18%.

**Figure 12:**
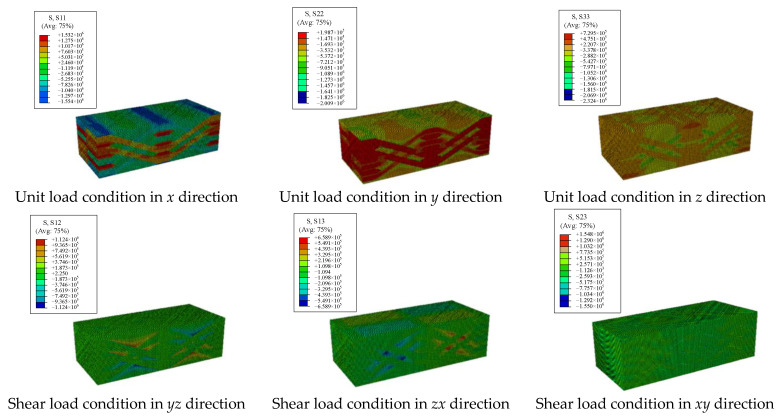
The stress distribution under different load boundary conditions.

The specific *x*, *y* and *z* axial tensile and *xy*, *yz*, *zx* axial shear loads were applied to the 2.5D braided composite meso model, and the statics analysis was carried out. Figure 12 shows the stress results under the boundary conditions of six loads.

The stress vectors in the constitutive equation are 1, respectively, by applying specific loading boundary conditions, i.e., the equation *F_i_*/*V* = 1. The equivalent stiffness matrix *C* in the constitutive equation is easily represented by elements in the strain component.

(10) $$C=\left[\begin{array}{cccccc} 3.809\times 10^{-12} & -9.406\times 10^{-13} & -1.086\times 10^{-12} & 0 & 0 & 0\\ -9.406\times 10^{-13} & 3.821\times 10^{-12} & -1.360\times 10^{-12} & 0 & 0 & 0\\ -1.086\times 10^{-12} & -1.360\times 10^{-12} & 1.337\times 10^{-11} & 0 & 0 & 0\\ 0 & 0 & 0 & 2.883\times 10^{-11} & 0 & 0\\ 0 & 0 & 0 & 0 & 2.253\times 10^{-11} & 0\\ 0 & 0 & 0 & 0 & 0 & 1.189\times 10^{-11} \end{array}\right]$$

When specific loading boundary conditions are applied to the mesoscopic model in finite element, the strain component can be expressed by the displacement calculated statically. According to Formulas (5)–(8), when all the elements in the stress component are 1, the equivalent elastic parameter is equal to 1/*ε_i_*, where *ε* = [*ε*_1_*^m^*, *ε*_2_*^m^*, *ε*_3_*^m^*, *γ^m^*_23_, *γ^m^*_31_, *γ^m^*_12_]. Table 10 shows the values of equivalent thermoelastic parameters.

#### 3.2.3. Equivalent Results of Yarn Path Uncertainty

**Figure 13:**
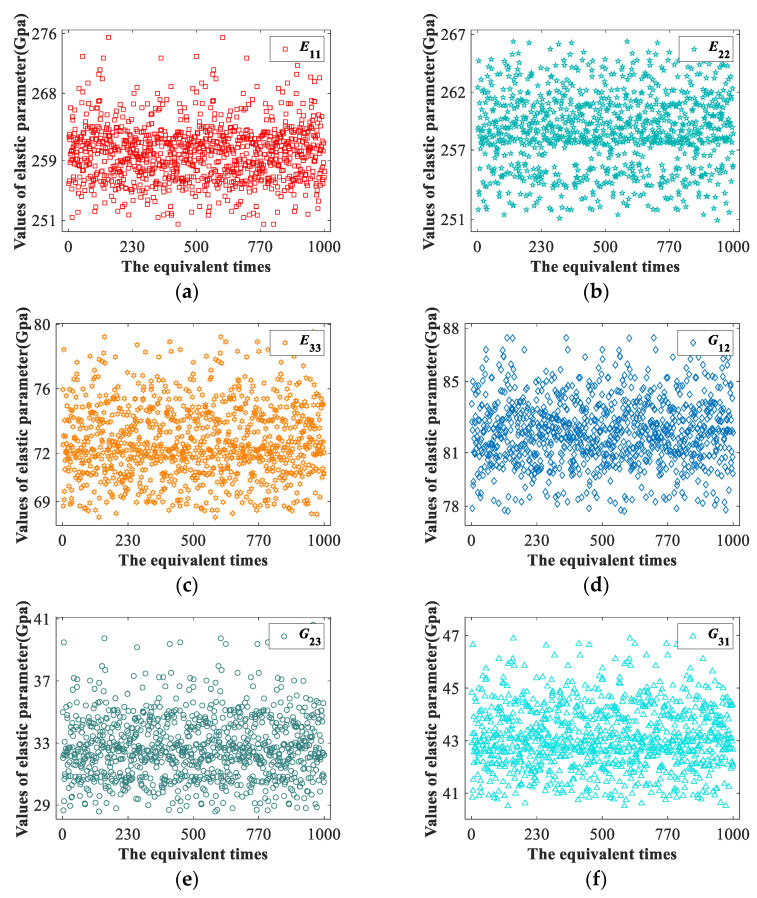
The scatter plots for equivalent elastic parameters. (**a**) Scatter diagram of *E*_11_ equivalent results. (**b**) Scatter diagram of *E*_22_ equivalent results. (**c**) Scatter diagram of *E*_33_ equivalent results. (**d**) Scatter diagram of *G*_12_ equivalent results. (**e**) Scatter diagram of *ν*_12_ equivalent results. (**f**) Scatter diagram of *α*_11_ equivalent results.

The center lines of two adjacent weft yarns of the 2.5D braided composite mesoscopic model change at [0,0.16], and the height difference between the weft yarns changes between [0,0.3]. Combined with the extreme values of the sample data and the mid-scale measurement in Table 11, it can be concluded that the yarn path uncertainty of 2.5D braided composite mainly has a relatively large impact on the elastic parameters *E*_11_, *E*_22_, *E*_33_, *G*_23_ and *G*_12_. Scatter diagrams of elastic parameters *E*_11_, *E*_22_, *E*_33_, *G*_12_, *G*_23_ and *G*_31_ with large standard deviations in Table 11 are shown in Figure 13.

According to the distribution of scattered points, the total distance R of the sample points of the above elastic parameters are 25.01 Gpa, 15.45 Gpa, 11.51 Gpa, 9.736 Gpa, 12.023 Gpa, and 6.695 Gpa, respectively.

Combined with the extreme values of sample data and the middle measurement in Table 11 and Table 12, it can be concluded that the yarn path uncertainty of 2.5D braided composite mainly has a relatively large impact on the elastic parameters *E*_11_, *E*_22_, *E*_33_, *G*_23_ and *G*_12_.

The correlation between elastic constant samples can be obtained from the data in Table 13. By analyzing the data in the table, it can be concluded that the main direction elastic modulus *E*_11_ is only relatively correlated with the parameters *ν*_12_ and *G*_12_ samples. At the same time, the correlation between *E*_22,_ *E*_33,_ *G*_12,_ *G*_23_ and *G*_31_ and the sample of parameter *ν*_12_ is small. The negative correlation between *ν*_23_, *ν*_31_ and other equivalent parameter sample data are consistent with the plain weave simulation example. The correlation between special *ν*_23_ and other equivalent parameter samples is negative, and the correlation between *E*_11_, *ν*_12_ parameter samples is less than 0.018.

## 4. Conclusions

An uncertainty modeling method is proposed in this paper for investigating the influence of randomness of the yarn path on mechanical properties, the statistical characteristics of the equivalent elastic and thermal parameters can be predicted. Assuming the yarn path is spatially varying in interval range, the variability of yarn path is represented geometrically in the unit cell of composite, and the Monte-Carlo simulation is adopted to obtain the variability of equivalent parameters. From the collection of the simulation data, it can be concluded that slight uncertainties of the yarn path of braided composite have a relatively impact on the elastic parameters and the thermal expansion coefficients, and the statistical correlation of elastic parameters is also obtained.

## Figures and Tables

**Table 1:** Periodic displacement boundary conditions.

Stiffness Coefficient		The Boundary of a Hexahedral Cell					
		* A *	* B *	* C *	* D *	* E *	* F *
The first column	[*ε*_1_*^m^*, 0, 0, 0, 0, 0]	(0, 0, 0)	(*x*_1_, 0, 0)	(_, 0, 0)	(_, 0, 0)	(_, 0, 0)	(_, 0, 0)
The second column	[0, *ε*_2_*^m^*, 0, 0, 0, 0]	(0, _, 0)	(0, _, 0)	(0, 0, 0)	(0, *y*_2_, 0)	(0, _, 0)	(0, _, 0)
The third column	[0, 0, *ε*_3_*^m^*, 0, 0, 0]	(0, 0, _)	(0, 0, _)	(0, 0, _)	(0, 0, _)	(0, 0, *z*_3_)	(0, 0, 0)
The fourth column	[0, 0, 0, *𝛾*_12_*^m^*, 0, 0]	(_, 0, 0)	(_, 0, 0)	(_, 0, 0)	(_, 0, 0)	(*y*_2_, 0, 0)	(0, 0, 0)
The fifth column	[0, 0, 0, 0, *𝛾*_23_*^m^*, 0]	(0, _, 0)	(0, _, 0)	(0, 0, 0)	(0, *z*_3_, 0)	(0, _, 0)	(0, _, 0)
The sixth column	[0, 0, 0, 0, 0, *𝛾*_13_*^m^*]	(0, 0, 0)	(0, 0, *x*_1_)	(0, 0, _)	(0, 0, _)	(0, 0, _)	(0, 0, _)

**Table 2:** Geometric design parameters of uncertainty model.

Parameter	Design Specification	Value
*J_L_*	Length of warp cross section	0.4
*W_L_*	Length of weft cross section	0.4
*G_L_*	Distance between warp and weft	[0.1,0.3]
*J_H_*, *W_H_*	Cross section thickness	0.056
*H_r1_*, *H_r2_*	Weft height changes	[0.082,0.111]
*H_m_*	Outer matrix height	0.04

**Table 3:** The material parameters of components.

	Elastic Modulus (*E*/Gpa)		Poisson’s Ratio		Shear Modulus (*G*/Gpa)	
	*E*_11_	*E*_22_	*ν*_12_	*ν*_23_	*G*_12_	*G*_23_
Carbon fiber	220	138	0.2	0.25	9	4.8
Matrix	350		0.3		140	

**Table 4:** The thermal expansion coefficients of components.

Thermal Expansion (10^−6^ k^−1^)	*α*_11_	*α*_22_	*α*_33_
Carbon fiber	−2 × 10^−7^	3 × 10^−6^	3 × 10^−6^
Matrix	6.5 × 10^−6^		

**Table 5:** Equivalent thermoelastic parameters.

*E*_11_ (Gpa)	*E*_22_ (Gpa)	*E*_33_ (Gpa)	*ν*_12_	*ν*_23_	*ν*_31_	*G*_12_ (Gpa)	*G*_23_ (Gpa)	*G*_31_ (Gpa)	*α*_11_ (10^−6^)	*α*_22_ (10^−6^)	*α*_33_ (10^−6^)
196.4	195.3	44.2	0.2	0.2	0.1	52.9	14.5	12.9	4.93 × 10^−6^	4.99 × 10^−6^	5.62 × 10^−6^

**Table 6:** Statistics of equivalent elastic parameters of 2D braided composites.

Statistic	*E*_11_ (*E*/Gpa)	*E*_22_ (*E*/Gpa)	*E*_33_ (*E*/Gpa)	*ν*_12_	*ν*_23_	*ν*_31_	*G*_12_ (*G*/Gpa)	*G*_23_ (*G*/Gpa)	*G*_31_ (*G*/Gpa)
Mean value	191.113	189.543	40.703	0.208	0.253	0.054	50.380	13.566	12.465
Standard deviation	8.498	8.367	5.440	0.007	0.006	0.005	3.704	1.031	0.982

**Table 7:** The statistics of thermal expansion coefficient of 2D materials.

Statistic	*α*_11_ (10^−6^)	*α*_22_ (10^−6^)	*α*_33_ (10^−6^)
Mean value	4.823 × 10^−6^	4.885 × 10^−6^	5.456 × 10^−6^
Standard deviation	1.924 × 10^−7^	1.912 × 10^−7^	2.370 × 10^−7^

**Table 8:** The correlation coefficients between equivalent parameters of 2D braided composites.

Coefficient	*E*_11_	*E*_22_	*E*_33_	*ν*_12_	*ν*_23_	*ν*_31_	*G*_12_	*G*_23_	*G*_31_
*E*_11_	1	0.864	0.715	0.924	0.309	0.539	0.952	0.865	0.893
*E*_22_		1	0.717	0.742	0.194	0.651	0.952	0.884	0.874
*E*_33_			1	0.467	−0.274	0.954	0.652	0.818	0.833
*ν*_12_				1	0.443	0.251	0.908	0.684	0.734
*ν*_23_		*sym*			1	−0.469	0.328	−0.018	0.237
*ν*_31_						1	0.515	0.765	0.706
*G*_12_							1	0.859	0.874
*G*_23_								1	0.806
*G*_31_									1

**Table 9:** The geometric design parameters of uncertainty model.

Parameter	Design Specification	Value
*J_H_*	Height of warp section	0.169
*J_L_*	Total length of warp section	1.015
*W_H_*	Height of weft cross section	0.169
*W_L_*	Length of weft cross section	0.6
*G_L_*	Distance between warp yarns	[1.535,1.835]
*H*_1_, *H*_2_	Weft height changes	[0.516,0.836]
*H_m_*	Outer matrix height	0.04

**Table 10:** The equivalent thermoelastic parameters.

*E*_11_ (Gpa)	*E*_22_ (Gpa)	*E*_33_ (Gpa)	*ν*_12_	*ν*_23_	*ν*_31_	*G*_12_ (Gpa)	*G*_23_ (Gpa)	*G*_31_ (Gpa)	*α*_11_ (10^−6^)	*α*_22_ (10^−6^)	*α*_33_ (10^−6^)
261.1	255.1	66.5	0.25	0.37	0.07	80.6	30.8	41.3	5.74 × 10^−6^	5.80 × 10^−6^	6.27 × 10^−6^

**Table 11:** Statistics of equivalent elastic parameters of 2.5D braided composites.

Statistic	*E*_11_ (E/Gpa)	*E*_22_ (E/Gpa)	*E*_33_ (E/Gpa)	*ν*_12_	*ν*_23_	*ν*_31_	*G*_12_ (G/Gpa)	*G*_23_ (G/Gpa)	*G*_31_ (G/Gpa)
Mean value	260.083	258.625	72.725	0.244	0.360	0.079	82.194	32.677	43.121
Standard deviation	3.864	3.223	2.238	0.003	0.003	0.002	1.860	2.015	1.229

**Table 12:** The 2.5D material thermal expansion coefficient statistics.

Statistic	*α*_11_ (10^−6^ k^−1^)	*α*_22_ (10^−6^ k^−1^)	*α*_33_ (10^−6^ k^−1^)
Mean value	5.734 × 10^−6^	5.804 × 10^−6^	6.265 × 10^−6^
Standard deviation	4.118 × 10^−8^	3.797 × 10^−8^	1.326 × 10^−8^

**Table 13:** The correlation coefficients between equivalent parameters of 2.5D braided composites.

Coefficient	*E*_11_	*E*_22_	*E*_33_	*ν*_12_	*ν*_23_	*ν*_31_	*G*_12_	*G*_23_	*G*_31_
*E*_11_	1	0.126	0.113	0.994	−0.018	−0.179	0.438	0.038	0.096
*E*_22_		1	0.958	0.126	−0.949	0.887	0.934	0.931	0.945
*E*_33_			1	0.093	−0.910	0.937	0.893	0.968	0.997
*ν*_12_				1	−0.020	−0.204	0.435	0.015	0.074
*ν*_23_		*sym*			1	−0.863	−0.839	−0.902	−0.897
*ν*_31_						1	0.734	0.959	0.948
*G*_12_							1	0.860	0.877
*G*_23_								1	0.975
*G*_31_									1

## Data Availability

Not applicable.
